# Protective Role of Spermidine Against Diabetes-Induced Ovarian and Endometrial Injury via LC3 and Beclin-1 Modulation

**DOI:** 10.3390/antiox14111294

**Published:** 2025-10-28

**Authors:** Bakiye Akbaş, Gülseren Dinç, Ahmet Akbaş, Nadir Adnan Hacım, Gülçin Ercan, Hatice Aygün, Oytun Erbaş

**Affiliations:** 1 Department of Obstetrics and Gynecology, Medical Faculty, Karadeniz Technical University, Trabzon 61080, Turkey; gulserendinc1@gmail.com; 2 Department of General Surgery, Faculty of Medicine, Karadeniz Technical University, Trabzon 61080, Turkey; draakbas@hotmail.com; 3 Department of General Surgery, Güneşli Erdem Hospital, Istanbul 34212, Turkey; adnanhcm@hotmail.com; 4 Department of General Surgery, Sultan 2. Abdulhamid Han Educational and Research Hospital, Istanbul Provincial Directorate of Health, Istanbul 34668, Turkey; ghepgul@hotmail.com; 5 Neuroscience Laboratory, Biruni University Research Center (BAMER), Biruni University, Istanbul 34000, Turkey; hatice_5aygun@hotmail.com; 6 Faculty of Medicine, Biruni University Research Center (BAMER), Biruni University, Istanbul 34000, Turkey; oytunerbas2012@gmail.com

**Keywords:** diabetes, spermidine, ovary, uterus, oxidative stress, autophagy, fibrosis, AMH

## Abstract

**Background:** Diabetes mellitus adversely affects female reproductive health by inducing oxidative stress, impairing autophagy, and promoting fibrotic remodeling in ovarian and uterine tissues. Spermidine, a natural polyamine, has gained attention as an antioxidant and autophagy enhancer. This study aimed to investigate the potential protective role of spermidine against diabetes-induced reproductive injury in rats. **Methods:** Thirty adult female Wistar rats were randomly divided into three groups (*n* = 10 each): Control, Diabetes, and Diabetes + Spermidine. Diabetes was induced with streptozotocin (60 mg/kg, i.p.). After confirmation of hyperglycemia (≥250 mg/dL), rats received either saline or spermidine (40 mg/kg/day, oral gavage) for four weeks. At sacrifice, plasma anti-Müllerian hormone (AMH) levels were determined, and ovarian and uterine tissues were assessed histologically and biochemically for oxidative stress markers (GSH, MDA, Nrf2), autophagy proteins (LC3, Beclin-1), and fibrosis indicators (TGF-β, histological scoring). **Results:** Diabetic rats exhibited severe hyperglycemia, pronounced follicular and endometrial degeneration, increased fibrosis, reduced plasma AMH, depleted GSH, SOD, CAT, GPx and Nrf2, and elevated MDA (*p* < 0.001). Spermidine treatment significantly mitigated these alterations, lowering glucose levels, alleviating histopathological injury, elevating the antioxidant defense (GSH, SOD, CAT, GPx) and the Nrf2 and decreasing MDA and TGF-β concentrations (*p* < 0.05 vs. Diabetes). Moreover, spermidine supplementation enhanced LC3 and Beclin-1 expression, suggesting improved autophagic activity. **Conclusions:** Spermidine counteracts diabetes-induced ovarian and uterine damage by reinforcing antioxidant defense, stimulating autophagy, and limiting fibrosis. These findings highlight spermidine as a promising adjunctive agent to support female reproductive health under diabetic conditions.

## 1. Introduction

Diabetes mellitus (DM) adversely affects the female reproductive system. Nearly 40% of diabetic women develop reproductive disorders such as delayed menarche, menstrual irregularities, infertility, polycystic ovary syndrome features, and premature menopause, at higher rates than in non-diabetic women [1]. Both type 1 and type 2 DM contribute through endocrine and metabolic disturbances (insulin deficiency or resistance with hyperinsulinemia) that impair gonadal function [1]. Beyond systemic effects, hyperglycemia directly alters ovarian and uterine structures in animal models [2,3].

Several mechanisms underlie diabetes-induced reproductive damage, mainly oxidative stress, autophagy disruption, and fibrosis. Persistent hyperglycemia increases reactive oxygen species (ROS), weakens antioxidant defenses, and accelerates advanced glycation end-product (AGE) formation [2]. These processes activate inflammatory and fibrogenic pathways, particularly TGF-β, leading to ovarian and uterine remodeling [2]. Although autophagy normally clears damaged organelles, chronic diabetic stress impairs its flux, worsening injury. Evidence shows that such dysregulation fosters fibrosis and tissue damage [4].

In diabetic models, ovaries show marked oxidative injury and functional decline. STZ-induced rats exhibit higher malondialdehyde (MDA) and follicular atresia, with reduced antioxidant enzymes (SOD, CAT) and corpora lutea [5]. Histology reveals cortical and medullary atrophy, widespread follicular degeneration, and stromal collagen accumulation [2], reflecting ROS-mediated loss and fibrotic remodeling.

The uterus is similarly vulnerable, displaying reduced endometrial volume and epithelial disruption [3]. Ultrastructural analyses show damaged cell borders, basement membranes, and thick, disorganized stromal collagen [3]. These fibrotic changes highlight oxidative stress–driven uterine injury. Antioxidant therapies such as curcumin preserve endometrial structure and prevent collagen bundling, underscoring oxidative stress and fibrosis as therapeutic targets [3].

Spermidine, a natural polyamine, has potent antioxidant, anti-inflammatory, and autophagy-promoting properties [6]. In ovaries, it enhances antioxidant enzymes (SOD, CAT), lowers lipid peroxidation (MDA), and reduces follicular atresia and apoptosis, while upregulating Beclin-1 and LC3 and decreasing p62, indicating restored autophagic flux [7]. Spermidine also attenuates fibrosis; in bleomycin-induced lung injury, it reduced collagen deposition and hydroxyproline via autophagy induction and stress relief [8]. Moreover, it activates Nrf2/HO-1 signaling, suppressing ROS accumulation and ferroptosis [9]. Through these combined actions—antioxidant defense, autophagy enhancement, and antifibrotic activity—spermidine may counteract diabetic reproductive organ damage.

Despite its promising properties, spermidine’s role in protecting diabetic female reproductive organs is largely unknown. No study has examined whether it counteracts ovarian and uterine damage through antioxidant, autophagy-regulating, and anti-fibrotic effects. This study addresses that gap by testing spermidine in STZ-induced diabetic rats, evaluating oxidative stress, autophagy, and fibrosis. We hypothesize that spermidine preserves reproductive tissues by reducing oxidative stress, restoring autophagy, and limiting fibrotic remodeling.

## 2. Material and Methods

### 2.1. Animals

A total of 30 adult female Wistar albino rats (10–12 weeks old, 150–200 g) were included in the present study. All procedures were conducted in compliance with the Guide for the Care and Use of Laboratory Animals (NIH, USA) and were approved by the Institutional Animal Ethics Committee (Ethical approval no: 8481526012, date: 21 October 2019). Animals were supplied by the Experimental Animal Center of Science University. During the experimental period, rats were kept in pairs in stainless steel cages under controlled environmental conditions (22 ± 2 °C; 12 h light/dark cycle) with free access to standard laboratory chow and water.

### 2.2. Experimental Protocol

#### 2.2.1. STZ-Induced Diabetes

Experimental diabetes was induced in 20 female rats by a single intraperitoneal injection of streptozotocin (STZ, 60 mg/kg; prepared in 0.9% NaCl and buffered to pH 4.0 with 0.2 M sodium citrate; Sigma-Aldrich, St. Louis, MO, USA). An additional 10 rats that did not receive STZ served as the non-diabetic control group. Fasting blood glucose values were determined from tail vein samples prior to injection (baseline). Twenty-four hours following STZ administration, glucose levels were reassessed using glucose oxidase reagent strips (Boehringer Mannheim, Indianapolis, IN, USA). Animals with fasting blood glucose ≥ 250 mg/dL were considered diabetic and included in the study, while those with levels < 120 mg/dL were assigned to the control group.

#### 2.2.2. Experimental Groups

Once diabetes was confirmed, the animals were randomly assigned into three experimental groups (*n* = 10 per group):

Group 1 (Control): Non-diabetic rats that received 1 mL/kg/day saline by oral gavage for four consecutive weeks.

Group 2 (Diabetes + Vehicle): Diabetic rats administered 1 mL/kg/day saline by oral gavage for four consecutive weeks.

**Figure 1:**
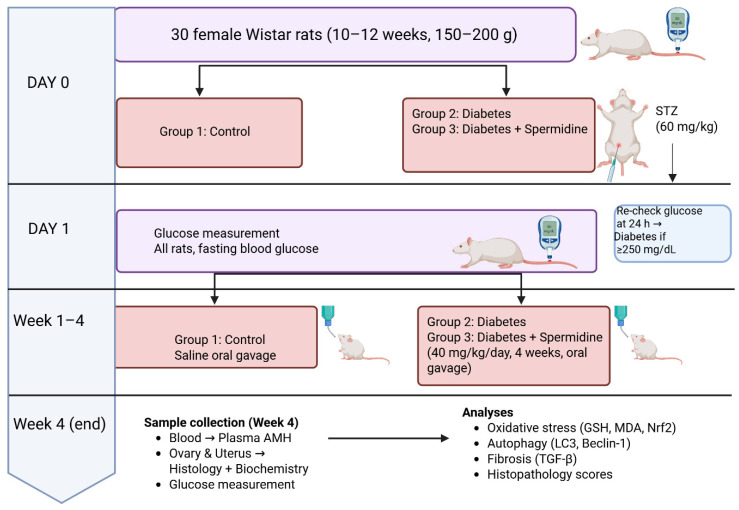
Experimental design of the study.

Group 3 (Diabetes + Spermidine): Diabetic rats treated with spermidine (40 mg/kg/day) by oral gavage for four consecutive weeks. (Figure 1)

#### 2.2.3. Sample Collection

At the end of the four-week treatment period, all animals were euthanized by cervical dislocation under deep anesthesia induced with ketamine (100 mg/kg; Ketasol, Richterpharma AG, Wels, Austria) and xylazine (50 mg/kg; Rompun, Bayer, Germany). Blood samples were obtained via cardiac puncture for biochemical analysis. The ovaries and uteri were immediately excised, cleaned of surrounding fat, and prepared for both histopathological and biochemical evaluations.

#### 2.2.4. Blood Glucose Measurement

Fasting blood glucose levels were determined from tail vein samples prior to STZ injection to establish baseline values. Twenty-four hours following STZ administration, glucose concentrations were reassessed using glucose oxidase reagent strips (Boehringer Mannheim, Indianapolis, IN, USA) to verify the induction of diabetes. Animals with fasting glucose levels ≥250 mg/dL were classified as diabetic and included in the study, whereas those with values below 120 mg/dL were designated as controls.

Thirty female Wistar rats (*n* = 10/group) were allocated to Control, Diabetes, and Diabetes + Spermidine groups. On Day 0, diabetes was induced in Groups 2–3 with STZ (60 mg/kg, i.p.); Group 1 served as non-diabetic controls. Blood glucose was measured at baseline and 24 h post-STZ (≥250 mg/dL = diabetic). For 4 weeks, Group 1 received saline, Group 2 remained untreated, and Group 3 received spermidine (40 mg/kg/day, oral). At Week 4, blood was collected for plasma AMH, and ovarian/uterine tissues were analyzed histologically and biochemically (oxidative stress, autophagy, fibrosis markers).

### 2.3. Histopathological Evaluation

For histological examination, uterine and ovarian tissues were fixed in 10% buffered neutral formalin, processed through paraffin embedding, and cut into 4-μm thick sections. All samples were stained with hematoxylin and eosin (H&E) and analyzed under an Olympus BX51 microscope equipped with a C-5050 digital camera (Olympus, Tokyo, Japan).

Uterus: Endometrial gland degeneration and stromal fibrosis were graded semi-quantitatively on a 0–3 scale, where 0 indicated normal histology, 1 represented mild alterations (<33% of the section), 2 denoted moderate involvement (33–66%), and 3 reflected severe injury (>66%).

Ovary: Follicular degeneration and stromal fibrosis were similarly assessed on a 0–3 scale, with 0 defined as intact morphology, 1 as mild degeneration (<33%), 2 as moderate alterations (33–66%), and 3 as severe damage involving more than two-thirds of the section.

The intensity of histopathological changes in H&E-stained sections was evaluated using a semi-quantitative scoring system by two independent, blinded histologists. Inter-observer agreement was assessed using Cohen’s weighted kappa (0–3 scale), which demonstrated substantial concordance (κ = 0.85, *p* < 0.001).

### 2.4. Measurement of Plasma AMH Levels

Following cardiac puncture at sacrifice, whole blood samples were collected into heparinized tubes and immediately centrifuged at 3000× *g* for 10 min at 4 °C to separate plasma. The obtained plasma fractions were carefully aliquoted and stored at −80 °C until biochemical analyses were performed. Plasma anti-Müllerian hormone (AMH) concentrations were quantified using a commercially available ELISA kit (Biosciences, Irvine, CA, USA). Prior to analysis, each sample was diluted at a ratio of 1:2 with the provided assay buffer to ensure that values fell within the linear detection range. The assay was carried out according to the manufacturer’s protocol: briefly, diluted samples and standards were added to microplate wells precoated with AMH-specific capture antibodies, followed by incubation with biotinylated detection antibodies and streptavidin–HRP conjugate. Following color development with the TMB substrate, absorbance was read at 450 nm using a microplate spectrophotometer. Plasma AMH levels were calculated from the standard curve and reported as ng/mL.

### 2.5. Biochemical Analysis of Ovarian and Uterine Tissues

#### 2.5.1. Tissue Preparation

Following euthanasia, the ovaries and uteri were promptly dissected, washed with ice-cold saline to eliminate blood residues, and stored at −20 °C until analysis. For biochemical evaluations, tissue samples were homogenized in five volumes of ice-cold phosphate-buffered saline (PBS, pH 7.4) using a glass–Teflon homogenizer. The homogenates were then centrifuged at 5000× *g* for 15 min at 4 °C, and the resulting supernatants were collected for subsequent assays. Total protein content was determined according to the Bradford method, employing bovine serum albumin (BSA) as the reference standard [10].

#### 2.5.2. Determination of Autophagy- and Fibrosis-Related Proteins (LC3, Beclin-1, TGF-β)

Levels of LC3, Beclin-1, and TGF-β in ovarian and uterine supernatants were determined using commercially available rat ELISA kits, applied according to the manufacturer’s instructions. Each sample was assayed in duplicate to ensure reproducibility. Absorbance values were recorded at 450 nm with a microplate spectrophotometer (Multiskan GO, Thermo Fisher Scientific, Waltham, MA, USA), and concentrations were calculated using standard calibration curves generated for each analyte.

#### 2.5.3. Measurement of Ovarian Nrf2 Levels

Quantification of ovarian Nrf2 concentrations was carried out with a rat-specific ELISA kit (catalog no.MBS752046, MyBioSource, San Diego, CA, USA). According to the manufacturer’s data, the assay exhibits high specificity for NFE2L2 without significant cross-reactivity to related proteins. The kit is validated for use in plasma, serum, cell culture supernatants, body fluids, and tissue homogenates, allowing reliable and reproducible detection of Nrf2 in rat ovarian homogenates.

#### 2.5.4. Determination of Tissue Glutathione (GSH) Levels

Reduced glutathione (GSH) was quantified as an index of antioxidant capacity. Ovarian tissues were homogenized in phosphate buffer (pH 7.5) at a ratio of 1:5 (*w*/*v*) under ice-cold conditions to preserve enzyme activity, and centrifuged at 3000× *g* for 5 min at 4 °C. The resulting supernatants were assayed spectrophotometrically based on a modified Ellman method. In this colorimetric assay, the sulfhydryl (-SH) groups of reduced glutathione (GSH) react with 5,5′-dithiobis-(2-nitrobenzoic acid) (DTNB), producing 5-thio-2-nitrobenzoic acid (TNB), which generates a yellow-colored chromophore. The intensity of this color, corresponding to TNB formation, was spectrophotometrically quantified at 412 nm, and GSH concentrations were subsequently calculated by reference to a standard calibration curve. GSH concentrations were calculated from a standard calibration curve and normalized to total protein content, with results expressed as nmol GSH per mg protein. All samples were measured in duplicate to minimize analytical variation.

#### 2.5.5. Determination of Antioxidant Enzyme Levels

Superoxide dismutase (SOD), catalase (CAT), and glutathione peroxidase (GPx) concentrations in ovarian and uterine tissue homogenates were measured using rat ELISA kits (SOD: AssayGenie, Cat. No. AEFI00956; CAT: MyBioSource, Cat. No. MBS453055; GPx: MyBioSource, Cat. No. MBS744364). Tissues were homogenized in ice-cold PBS (pH 7.4) and centrifuged at 12,000× *g* for 10 min at 4 °C. The supernatants were assayed in duplicate according to the manufacturer’s protocols. Absorbance was read at 450 nm using a microplate spectrophotometer (Multiskan GO, Thermo Fisher Scientific, Waltham, MA, USA), and concentrations were calculated from standard calibration curves. The results were normalized to total protein content (BCA assay) and expressed as ng/mg protein.

#### 2.5.6. Assessment of Lipid Peroxidation (MDA Levels)

Malondialdehyde (MDA), a major end-product of lipid peroxidation, was quantified using the thiobarbituric acid reactive substances (TBARS) method. Briefly, tissue homogenates were mixed with trichloroacetic acid and thiobarbituric acid (TBA) reagents, vortexed, and subsequently incubated at 100 °C for 60 min. After cooling on ice, the mixtures were centrifuged at 3000 rpm for 20 min. The absorbance of the supernatants was measured at 535 nm using a UV–visible spectrophotometer. MDA concentrations were calculated against a calibration curve prepared with 1,1,3,3-tetraethoxypropane and normalized to the total protein content. The results were expressed as nmol MDA per mg protein.

### 2.6. Statistical Analysis

All statistical analyses were performed using IBM SPSS Statistics, version 25.0 (IBM Corp., Armonk, NY, USA). Data distribution was assessed using the Shapiro–Wilk test for normality, and homogeneity of variances was evaluated by Levene’s test. For parameters meeting parametric assumptions, one-way analysis of variance (ANOVA) was applied, followed by Tukey’s post hoc test for pairwise comparisons when variances were homogeneous, or Tamhane’s T2 test when heterogeneity of variances was present. The results of parametric variables are expressed as mean ± standard error of the mean (SEM).

For data not fulfilling parametric assumptions, the Kruskal–Wallis test was employed. When significant differences were detected, pairwise comparisons were performed using the Mann–Whitney U test with Bonferroni correction to adjust for multiple testing. Non-parametric data are presented as median [interquartile range, IQR].

A *p*-value < 0.05 was considered statistically significant for all analyses. Graphical presentations were generated using GraphPad Prism, version 9.0 (GraphPad Software/San Diego-CA-USA).

## 3. Results

### 3.1. Blood Glucose Levels (mg/dL)

**Figure 2:**
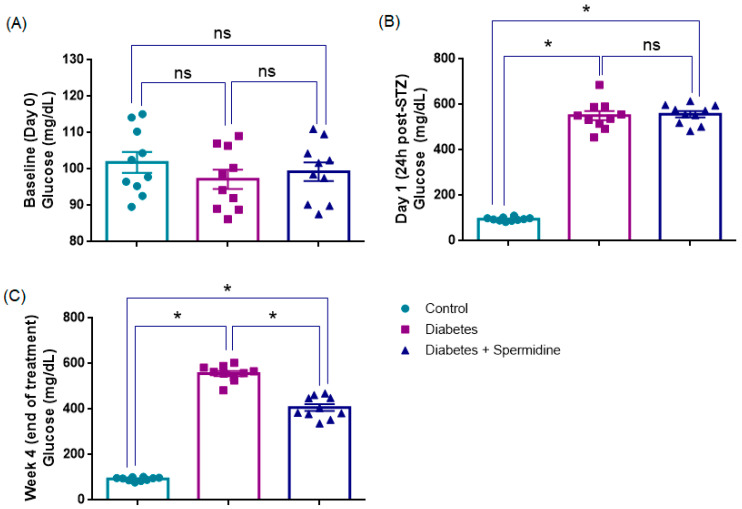
Comparative blood glucose concentrations in control, diabetic, and spermidine-treated diabetic groups. (**A**) Baseline values (Day 0), (**B**) glucose levels at 24 h after STZ administration, and (**C**) values recorded at the end of the 4-week treatment period. The results are expressed as mean ± SEM. Statistical evaluation was performed using one-way ANOVA followed by appropriate post hoc tests. * *p* < 0.001; ns: not significant.

At baseline (Day 0), blood glucose values were comparable among the three groups, with no significant differences observed (*p* > 0.05), confirming similar metabolic status prior to diabetes induction (Table 1, Figure 2).

Twenty-four hours after STZ injection, both the Diabetes and Diabetes + Spermidine groups exhibited a sharp increase in blood glucose compared to the Control group (*p* < 0.001), confirming successful induction of diabetes. Glucose levels did not differ between diabetic animals receiving vehicle or spermidine at this early time point (*p* > 0.05) (Table 2, Figure 1).

At the end of the 4-week treatment, glucose levels remained markedly elevated in diabetic rats compared to controls (*p* < 0.001). However, spermidine administration significantly reduced hyperglycemia relative to untreated diabetics (*p* < 0.001), although values did not fully normalize to control levels (*p* < 0.001) (Table 2, Figure 1).

### 3.2. Histopathological Scores and Plasma AMH Levels

**Figure 3:**
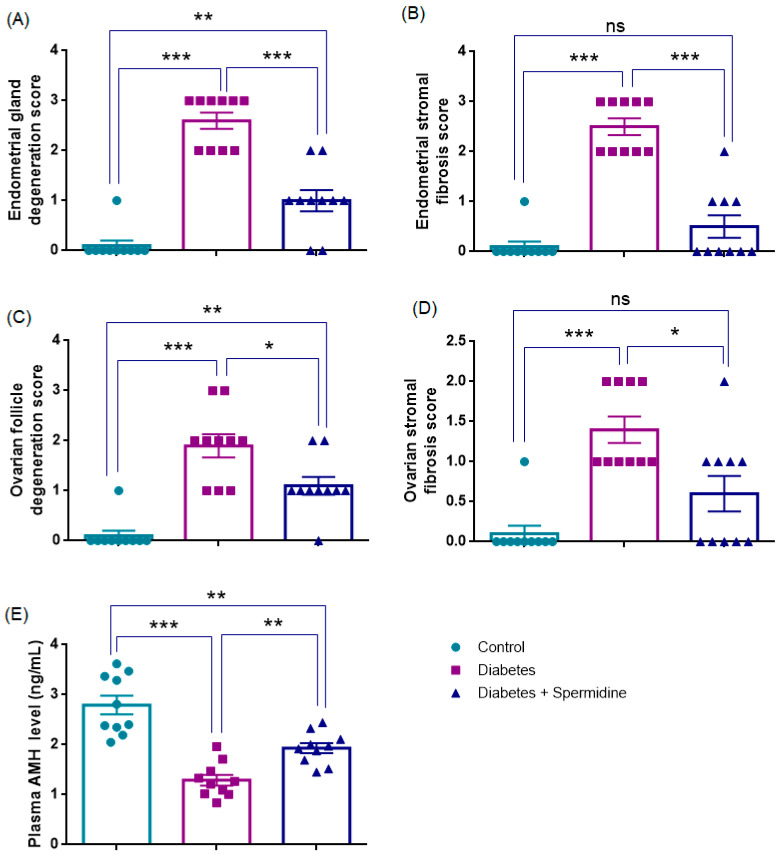
Histopathological degeneration scores, fibrosis scores, and plasma AMH levels in control, diabetic, and spermidine-treated diabetic rats. (**A**) Endometrial gland degeneration, (**B**) Endometrial stromal fibrosis, (**C**) Ovarian follicle degeneration, (**D**) Ovarian stromal fibrosis, and (**E**) Plasma AMH concentrations. Data are expressed as mean ± SEM. * *p* < 0.05, ** *p* < 0.01, *** *p* < 0.001. ns: not significant.

Histopathological evaluation revealed that endometrial gland degeneration was significantly increased in the Diabetes group compared with Controls (*p* < 0.001). Spermidine treatment markedly reduced degeneration (*p* < 0.001), although values remained higher than in Controls (*p* < 0.01). Endometrial stromal fibrosis showed a similar pattern, being significantly elevated in diabetic rats versus Controls (*p* < 0.001), while spermidine treatment effectively reduced fibrosis (*p* < 0.001) to levels not different from Controls (*p* > 0.05) (Figure 3, Table 2).

Ovarian follicle degeneration was also significantly higher in Diabetes compared with Controls (*p* < 0.001). Spermidine administration significantly reduced this degeneration (*p* < 0.05), although values did not fully return to Control levels (*p* < 0.01). Likewise, ovarian stromal fibrosis was elevated in the Diabetes group compared with Controls (*p* < 0.001). Remarkably, spermidine treatment led to a pronounced reduction in fibrosis (*p* < 0.05), with values no longer significantly different from those of the Control group (*p* > 0.05) suggesting a near-complete restoration of stromal architecture (Figure 3, Table 2).

Consistent with these morphological findings, plasma AMH levels were significantly reduced in the Diabetes group versus Controls (*p* < 0.001). Spermidine treatment increased AMH compared with Diabetes (*p* < 0.01), although levels remained lower than Controls (*p* < 0.01) (Figure 3, Table 2).

#### 3.2.1. Figure 4 (Histopathology–Ovary, H&E Staining)

Histological examination of ovarian sections from the Control group (A–B) revealed a normal stromal architecture with clearly distinguishable primary, secondary, and tertiary follicles as well as well-preserved corpus luteum structures. In contrast, ovarian sections from the Diabetes + Saline group (C–D) demonstrated marked pathological alterations, including stromal fibrosis (*) and pronounced follicular degeneration (fd). These degenerative changes were characterized by disruption of stromal integrity and reduced follicular density. Notably, ovaries from the Diabetes + Spermidine group (E–F) exhibited a marked attenuation of these pathological changes, with decreased stromal fibrosis (*) and reduced follicular degeneration (f), indicating a partial preservation of ovarian tissue morphology.

**Figure 4:**
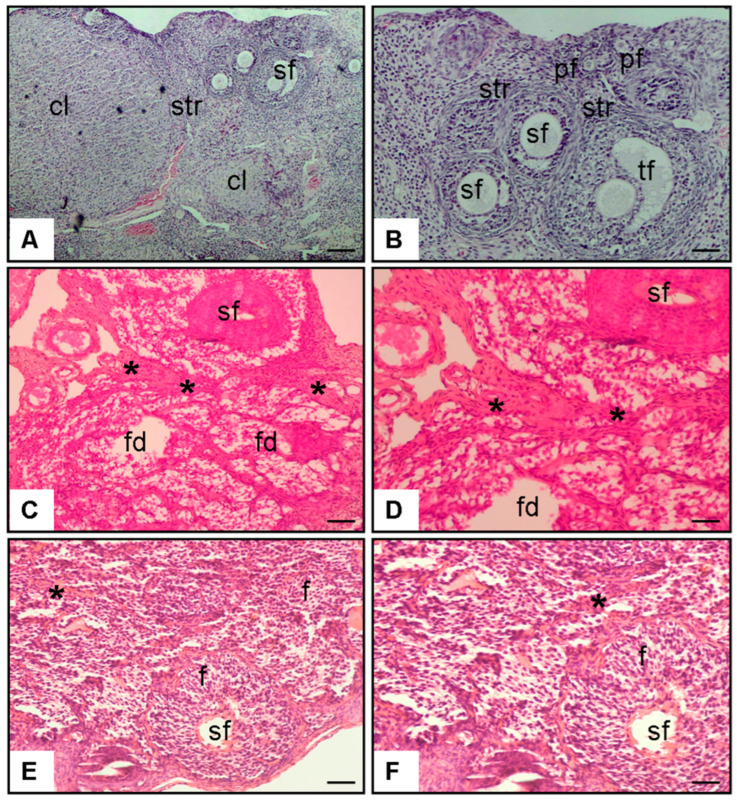
Representative H&E staining of ovarian sections at ×20 and ×40 magnification. (**A**,**B**) Control rats exhibited normal stromal organization (str), with clearly distinguishable primary (pf), secondary (sf), and tertiary follicles (tf), as well as preserved corpus luteum structures (cl). (**C**,**D**) Diabetic rats receiving saline displayed marked histopathological changes, including stromal fibrosis (*) and evident follicular degeneration (fd). (**E**,**F**) Spermidine-treated diabetic rats showed reduced stromal fibrosis (*) and attenuation of follicular degeneration (f), indicating partial protection of ovarian architecture. Representative ovarian tissue sections under 10× (**A**,**B**) and 20× (**C**–**F**) magnification. For digital scale calibration, 1 cm in the image corresponds to 25 µm at 10× and 12.5 µm at 20× magnification.

#### 3.2.2. Figure 5 (Histopathology–Uterus, H&E Staining)

Histological analysis of uterine sections from the Control group (A–B) showed a normal uterine cavity (C), intact endometrial glands (G), and well-organized smooth muscle layers (m). In contrast, sections from the Diabetes + Saline group (C–D) demonstrated extensive stromal fibrosis (*) and a marked reduction in the number and structural integrity of endometrial glands. These pathological changes were characterized by disrupted stromal architecture and thinning of the endometrial layer. Notably, in the Diabetes + Spermidine group (E–F), stromal fibrosis (*) was reduced and the number of endometrial glands (G) was preserved or increased compared to the untreated diabetic group, indicating a partial histological recovery of uterine morphology.

**Figure 5:**
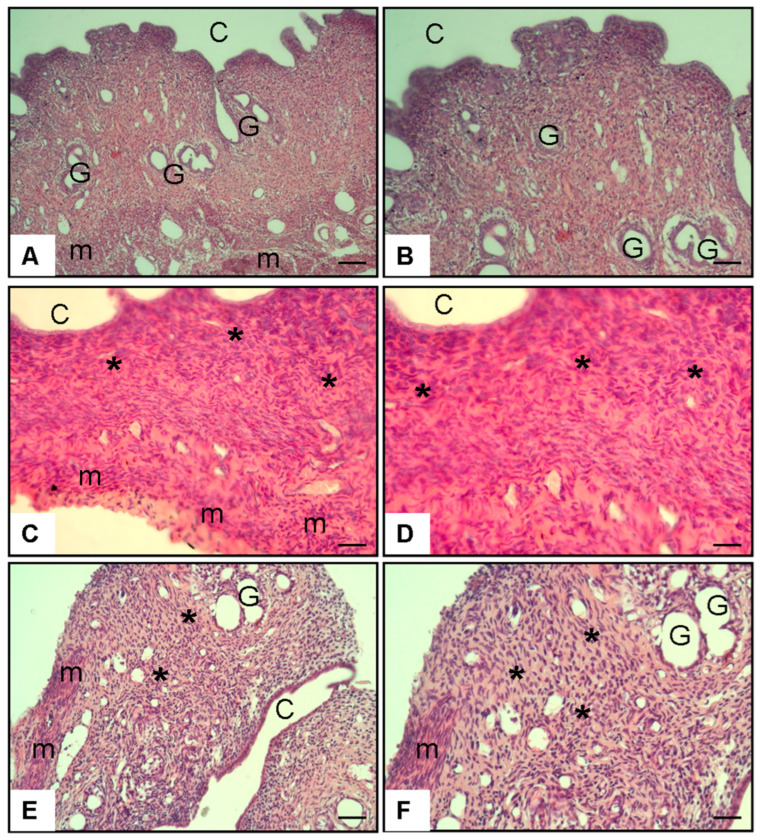
H&E staining of uterine sections from experimental groups at ×20 and ×40 magnification. (**A**,**B**) Control animals displayed normal uterine morphology with intact cavity (C), preserved endometrial glands (G), and organized smooth muscle layers (m). (**C**,**D**) Diabetic rats receiving saline showed marked pathological changes, including stromal fibrosis (*) and reduced endometrial gland density. (**E**,**F**) Spermidine-treated diabetic rats exhibited attenuation of stromal fibrosis (*) and partial restoration of endometrial glands (G), indicating a protective effect of treatment. Representative uterine tissue sections under 10× (**A**,**B**) and 20× (**C**–**F**) magnification. For digital scale calibration, 1 cm in the image corresponds to 25 µm at 10× and 12.5 µm at 20× magnification.

### 3.3. Oxidative Stress Parameters in Ovarian Tissue

**Figure 6:**
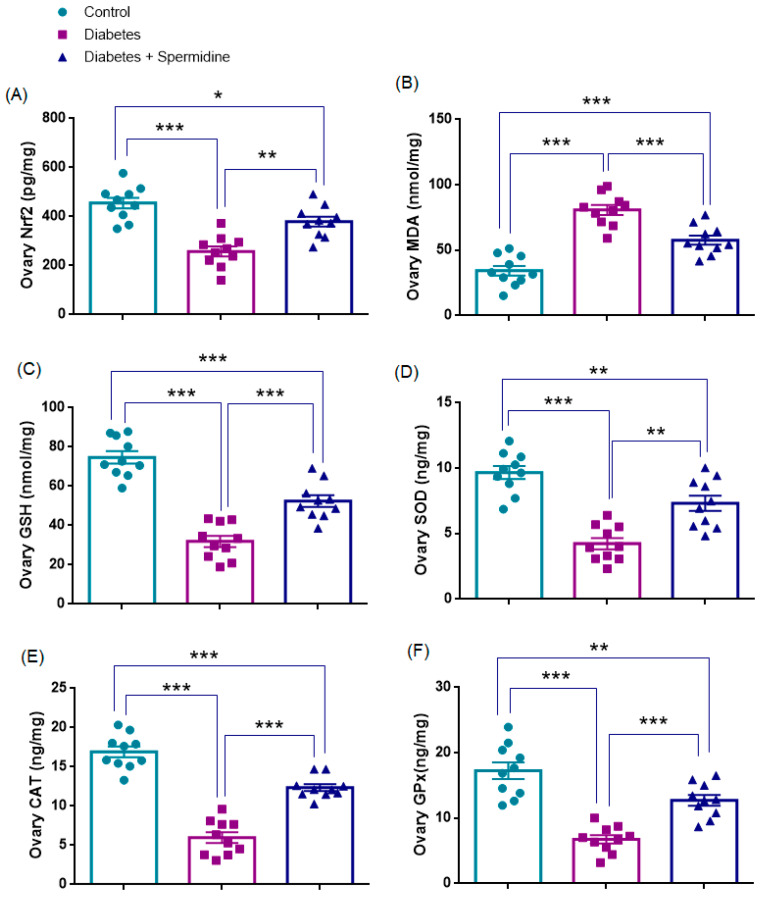
Ovarian oxidative stress parameters in Control, Diabetes, and Diabetes + Spermidine groups. Levels of (**A**) Nrf2, (**B**) MDA, (**C**) GSH, (**D**) SOD, (**E**) CAT, and (**F**) GPx are shown. Data are expressed as mean ± SEM. * *p* < 0.05, ** *p* < 0.01, *** *p* < 0.001.

The results of the analyses of oxidative stress parameters in ovarian homogenates are presented in Figure 6. Nrf2 and GSH concentrations were significantly reduced in the Diabetes group compared with Controls (all *p* < 0.001), whereas spermidine treatment significantly increased both parameters compared with Diabetes (*p* < 0.01; *p* < 0.001, respectively), although values remained below those of Controls (*p* < 0.05, *p* < 0.001, respectively).

Similarly, SOD, CAT, and GPx activities were significantly decreased in Diabetes compared with Controls (all *p* < 0.001), while spermidine significantly improved these enzyme activities relative to the Diabetic group (*p* < 0.01; *p* < 0.001; *p* < 0.001, respectively), although values did not completely return to Control levels (*p* < 0.01; *p* < 0.001; *p* < 0.01, respectively).

By contrast, MDA levels were significantly elevated in the Diabetes group relative to Controls (*p* < 0.001), and spermidine significantly reduced these values (*p* < 0.001), yet they still remained higher than in Controls (*p* < 0.001) (Figure 6, Table 3).

### 3.4. Oxidative Stress Parameters in Uterine Tissue

**Figure 7:**
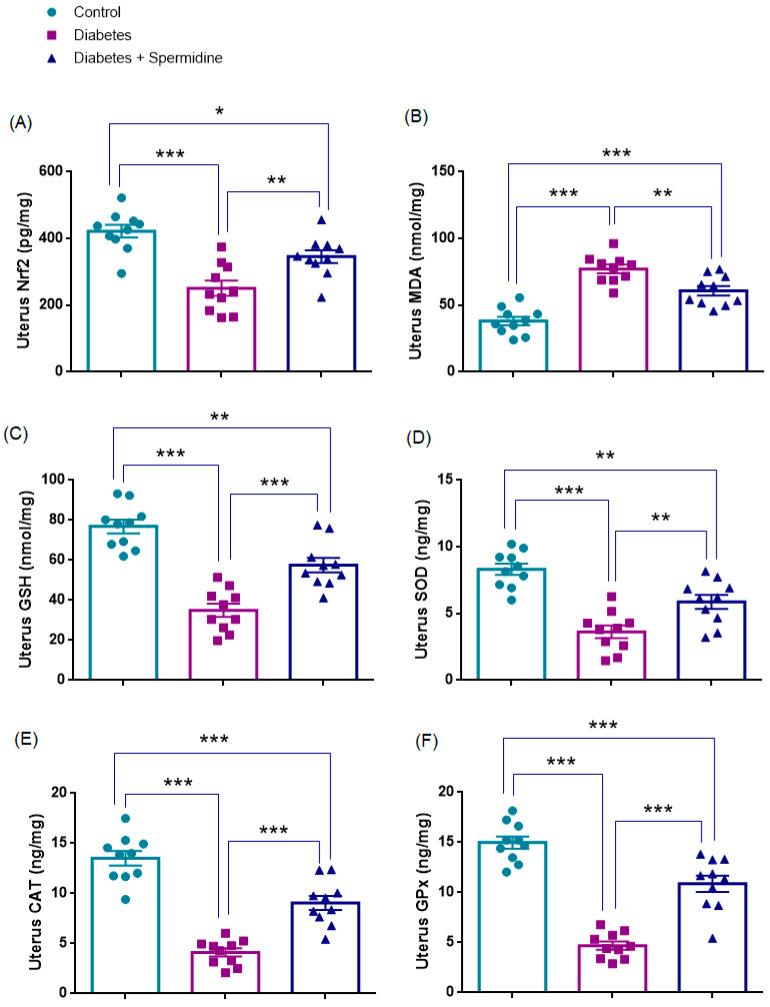
Uterine oxidative stress parameters in Control, Diabetes, and Diabetes + Spermidine groups. Levels of (**A**) Nrf2, (**B**) MDA, (**C**) GSH, (**D**) SOD, (**E**) CAT, and (**F**) GPx are shown. Data are expressed as mean ± SEM. * *p* < 0.05, ** *p* < 0.01, *** *p* < 0.001.

The results of the analyses of oxidative stress parameters in uterine homogenates are presented in Figure 7. Nrf2 expression and GSH concentrations were significantly reduced in the Diabetes group compared with Controls (all *p* < 0.001), whereas spermidine treatment significantly increased both parameters compared with Diabetes (*p* < 0.01; *p* < 0.001, respectively), although values remained below those of Controls (*p* < 0.05; *p* < 0.01, respectively).

Similarly, SOD, CAT, and GPx activities were significantly decreased in Diabetes compared with Controls (all *p* < 0.001), while spermidine significantly improved these enzyme activities relative to the untreated diabetic group (*p* < 0.01; *p* < 0.001; *p* < 0.001, respectively), although values did not completely return to control levels (*p* < 0.01; *p* < 0.001; *p* < 0.001, respectively).

By contrast, MDA levels were significantly elevated in the Diabetes group relative to Controls (*p* < 0.001), and spermidine significantly reduced these values compared with Diabetes (*p* < 0.01), yet they still remained higher than in Controls (*p* < 0.001) (Figure 7, Table 4).

### 3.5. Autophagy- and Fibrosis-Related Parameters in Ovarian Tissue

**Figure 8:**
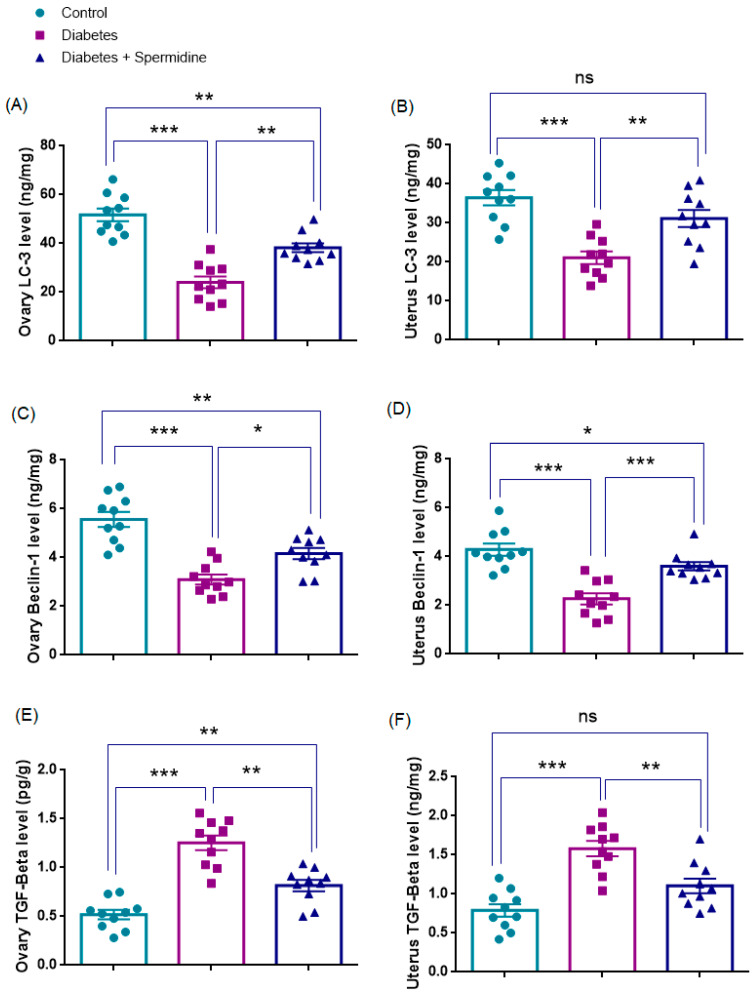
Effects of spermidine treatment on autophagy- and fibrosis-related markers in ovary and uterus tissues of diabetic rats. (**A**,**B**) LC3 levels in ovary and uterus; (**C**,**D**) Beclin-1 levels in ovary and uterus; (**E**,**F**) TGF-β levels in ovary and uterus. Data are presented as mean ± SEM. ns = not significant. * *p* < 0.05, ** *p* < 0.01, *** *p* < 0.001 versus indicated groups.

The results of the analyses of autophagy- and fibrosis-related parameters in ovarian homogenates are presented in Figure 8. Both LC3 and Beclin-1 levels were significantly reduced in the Diabetes group compared with Controls (*p* < 0.001 for both), indicating impaired autophagic activity. Spermidine treatment significantly increased LC3 (*p* < 0.01) and Beclin-1 (*p* < 0.05) compared with the Diabetes group, yet values for both markers remained lower than in Controls (*p* < 0.01 for each).

By contrast, TGF-β levels were significantly elevated in the Diabetes group compared with Controls (*p* < 0.001), reflecting enhanced profibrotic signaling, and spermidine significantly reduced TGF-β compared with Diabetes (*p* < 0.01), although values remained higher than in Controls (*p* < 0.01), indicating an incomplete attenuation of fibrosis (Figure 8, Table 5).

### 3.6. Autophagy- and Fibrosis-Related Parameters in Uterine Tissue

The results of the analyses of autophagy- and fibrosis-related parameters in uterine homogenates are presented in Figure 8. LC3 and Beclin-1, the key indicators of autophagic activity, were significantly reduced in the Diabetes group compared with Controls (both *p* < 0.001), indicating a reduction in autophagic activity. Spermidine treatment significantly increased LC3 (*p* < 0.01) and Beclin-1 (*p* < 0.001) compared with the untreated Diabetic group, suggesting a partial reactivation of the autophagic process. After treatment, LC3 levels did not differ from Controls (*p* > 0.05), pointing to near normalization, whereas Beclin-1 remained significantly lower (*p* < 0.05).

By contrast, TGF-β levels were significantly elevated in the Diabetes group compared with Controls (*p* < 0.001), consistent with enhanced profibrotic signaling. Spermidine administration significantly reduced TGF-β compared with Diabetes (*p* < 0.01), yet levels did not differ significantly from Controls (*p* > 0.05) (Figure 8, Table 5).

## 4. Discussion

This study shows that spermidine protects against STZ-induced reproductive injury by improving glycemia, preserving ovarian and uterine structure, reducing fibrosis, and maintaining AMH. Biochemically, it enhanced GSH and Nrf2, lowered MDA, and upregulated autophagy markers (LC3, Beclin-1) while suppressing TGF-β. These findings indicate antioxidant, autophagy-promoting, and antifibrotic actions, consistent with its systemic benefits [7,9,11,12,13]. To our knowledge, this is the first report showing spermidine modulates oxidative stress, autophagy, and TGF-β–mediated fibrosis in the diabetic ovary and uterus.

STZ administration induced severe hyperglycemia in female rats (fasting glucose > 500 vs. ~90–100 mg/dL baseline), consistent with previous models [14,15]. Spermidine treatment for four weeks significantly lowered glucose compared to untreated controls. This agrees with reports that spermidine modulates glucose homeostasis in rodents: elevated endogenous spermidine protects against diet-induced diabetes [16], supplementation prevented HbA1c increases in diabetic rats [17], and improved insulin resistance in high-fat diet mice [18]. Human studies also support these effects: higher intake is linked to lower glucose, insulin, and HOMA-IR [18], and serum spermidine inversely correlates with type 2 diabetes risk [11,19]. Collectively, spermidine enhances glycemic control and insulin sensitivity, which likely contributed to improved reproductive outcomes in our treated rats.

Untreated diabetic rats exhibited severe ovarian and uterine damage. Ovarian tissues showed widespread follicular atresia, stromal fibrosis, and loss of corpora lutea, with a marked decline in circulating AMH, indicating diminished ovarian reserve. Uteri displayed glandular loss, endometrial thinning, and collagen deposition. These findings are consistent with reports that diabetes accelerates follicular atresia and granulosa apoptosis [20], reduces ovarian reserve and alters endometrial morphology [21,22], and lowers AMH in STZ models [23]. Clinically, women with diabetes often show subfertility and accelerated ovarian aging, with meta-analyses confirming significantly reduced AMH in type 1 diabetes [24,25,26]. In our model, serum AMH was higher in spermidine-treated rats, suggesting partial preservation of ovarian reserve, although still below non-diabetic controls. Mechanistically, poor glycemic control disrupts gonadotropin signaling and promotes oxidative injury in reproductive tissues, thereby impairing folliculogenesis and endometrial receptivity. Spermidine therapy attenuated these diabetic lesions. Treated rats preserved follicles, corpora lutea, uterine glands, and endometrial thickness, with significantly less collagen than untreated diabetics. This improvement likely reflects reduced granulosa loss and apoptosis.

Severe hyperglycemia in our STZ-diabetic rats also led to systemic oxidative stress, as evidenced by elevated ovarian MDA (malondialdehyde) and depleted GSH, SOD, CAT, GPx consistent with oxidative damage. Many studies indicated that severe hyperglycemia in STZ-diabetic rats established glucotoxicity, as excess glucose promotes oxidative metabolism [27,28]. Chronic high glucose is known to generate excess reactive oxygen species (ROS) via auto-oxidation and advanced glycation, overwhelming antioxidant defenses and impairing redox regulators like Nrf2 [26,27,29]. We observed that diabetic ovaries had significantly lower Nrf2 expression, indicating suppression of this master antioxidant response pathway, which concurs with reports of downregulated Nrf2 signaling in diabetes-related organ damage [29]. Spermidine treatment in this model normalized redox status, characterized by enhanced Nrf2 expression, elevated GSH, GSH, SOD, CAT, GPx and decreased MDA levels approaching those of non-diabetic controls. Previous studies have shown that spermidine reactivates endogenous antioxidant pathways—likely by stabilizing Nrf2—thereby promoting the transcription of antioxidant genes [29]. Niu et al. [9] similarly demonstrated that spermidine activates the Nrf2/HO-1/GPX4 axis in ovarian tissue, thereby reducing ROS and preventing ferroptosis. Mechanistically, spermidine may stabilize Nrf2, enhancing antioxidant gene transcription and redox homeostasis.

Importantly, our findings demonstrate that STZ-induced diabetes disrupts the fine equilibrium between autophagy and fibrosis in reproductive tissues, whereas spermidine treatment appears to partially restore this balance. In untreated diabetic rats, ovarian levels of LC3 and Beclin-1 were markedly reduced, consistent with impaired autophagy. This observation is in line with earlier studies showing that STZ-induced diabetes suppresses autophagic activity in diverse organs—including myocardium, retina, immune cells, skeletal muscle, and ovaries—highlighting that hyperglycemia broadly impairs autophagy in metabolically active tissues [30,31]. Mechanistic studies further indicate that hyperglycemia hyperactivates nutrient-sensing mTORC1 and induces S-nitrosation of autophagy-related proteins, thereby blocking autophagic flux and limiting lysosomal clearance, while restoration of autophagy can attenuate collagen accumulation and fibrosis [32,33,34,35].

In contrast, spermidine has been widely recognized as a potent autophagy inducer through its ability to inhibit acetyltransferases and modulate mTOR signaling [36]. Experimental data across multiple organs—including ovary, liver, lung, kidney, and heart—demonstrate that spermidine supplementation consistently elevates autophagy markers such as Beclin-1 and LC3-II [8,29,37]. Notably, Jiang et al. [7] reported that three months of spermidine feeding increased ovarian Beclin-1 and LC3 levels while reducing p62/SQSTM1, thereby confirming activation of autophagic flux. Consistently, in our study, spermidine treatment significantly upregulated LC3 and Beclin-1 expression in diabetic ovaries, suggesting a reactivation of autophagy. By restoring autophagic activity, spermidine likely facilitates the clearance of damaged mitochondria and misfolded proteins, thereby alleviating cellular stress and reducing pro-apoptotic signaling.

In this study, TGF-β expression was significantly elevated in diabetic rats, consistent with histological fibrosis. As a key fibrogenic cytokine, TGF-β drives matrix deposition, and diabetes is known to upregulate it in reproductive and other tissues [22]. Deficient autophagy and excessive TGF-β create a vicious cycle: impaired clearance promotes profibrotic signaling, while TGF-β further suppresses autophagy [8,38]. Spermidine treatment reduced ovarian and uterine TGF-β, consistent with reports that polyamines enhance autophagy and attenuate fibrosis [39,40]. This antifibrotic effect is partly indirect via reduced oxidative stress and partly direct through TGF-β/Smad interference. Spermidine and spermine have inhibited TGF-β–driven fibrogenesis in multiple models [8,29], including bleomycin-induced lung fibrosis, where spermidine promoted autophagy and limited ER-stress apoptosis [8], and kidney fibrosis, where it suppressed TGF-β1 and collagen via Nrf2 activation [29]. Such dual regulation likely underlies the improved ovarian and uterine integrity observed here. Human studies also associate higher dietary spermidine intake with healthier cardiovascular and reproductive aging profiles [12,41], supporting its translational promise.

In STZ-induced diabetes models, the autophagic response exhibits a biphasic pattern; it increases adaptively during the early phase [42,43,44] but becomes markedly suppressed in metabolically active organs such as the kidney, heart, and ovary after 2–4 weeks [31,45]. In our study, the decrease in LC3 and Beclin-1 levels accompanied by an elevation in TGF-β at week 4 indicates suppression of autophagy. These findings suggest that the protective effects of spermidine may be associated with both improved glycemic control and the reactivation of cellular defense mechanisms. The reduction in glucose levels may have partially alleviated the inhibitory effect of hyperglycemia on autophagy [46,47,48], while the concurrent activation of Nrf2, increased antioxidant enzyme levels (SOD, CAT, GPx, GSH), and decreased MDA concentrations indicate the restoration of redox balance [7]. Thus, spermidine may have supported cellular repair by enhancing Nrf2-mediated antioxidant responses and rebalancing the autophagy–TGF-β axis. The observed increase in AMH may reflect both the indirect contributions of glycemic control and the potential direct regulatory effects of these mechanisms. Furthermore, considering that four weeks of hyperglycemia in rats roughly corresponds to approximately 2.5 years of human life [49,50], these findings suggest that spermidine may also exert beneficial effects during the transition to a chronic-like phase of diabetes.

Spermidine is a natural dietary polyamine, present in foods and produced by gut microbiota, and has gained attention as a geroprotective agent. Human studies associate higher intake with reduced mortality and improved cardiovascular outcomes, while early clinical trials confirm a favorable safety profile and cognitive benefits in older adults [12,51]. These characteristics suggest spermidine could be a practical adjunct therapy. Our findings indicate potential relevance for diabetic women, where supplementation might help preserve ovarian reserve and enhance endometrial receptivity. Future clinical trials are required to establish efficacy and dosing in this context, but the present data provide a mechanistic basis for translational exploration.

### Limitations

This study has several limitations. First, it was performed in a short-term STZ-induced type 1 diabetes model, which may not fully reflect the chronic and multifactorial context of human type 2 diabetes. Second, only a single spermidine dose and treatment duration were evaluated, restricting dose–response and long-term interpretations. Third, the study included only female rats, leaving potential sex-related differences unexplored.

In addition, spermidine was administered only after diabetes confirmation; therefore, the findings reflect post-treatment effects rather than prophylactic efficacy. Tissue spermidine levels in the ovary and uterus were not measured, preventing verification of tissue uptake. Future studies should evaluate prophylactic administration and tissue levels.

Finally, key mechanistic markers such as autophagic flux (p62/SQSTM1, lysosomal activity) and direct fibrosis measures (collagen content, Smad signaling) were not assessed, which limits mechanistic depth. Collectively, these factors constrain the translational strength of our findings, and future studies should address these aspects to enhance mechanistic insight and clinical relevance.

## 5. Conclusions

Our study demonstrated that STZ-induced diabetes caused significant reproductive injury in female rats, characterized by oxidative stress, impaired autophagy, enhanced TGF-β activity, and subsequent fibrosis and apoptotic changes leading to ovarian failure. Spermidine administration effectively counteracted these alterations by reducing oxidative stress, reactivating autophagic pathways, and limiting fibrotic remodeling. As a result, ovarian reserve markers such as AMH, follicular structures, and endometrial integrity were partially preserved. These findings suggest that spermidine exerts protective effects on both the ovary and uterus in the diabetic setting and may represent a promising candidate for preventing diabetes-related infertility.

## Figures and Tables

**Table 1:** Blood glucose levels in control, diabetic, and spermidine-treated diabetic rats.

	Control	Diabetes	Diabetes + Spermidine
Baseline blood glucose (mg/dL, Day 0)	102 ± 3	97 ± 2	99 ± 2
Blood glucose at 24 h post-STZ (mg/dL)	96 ± 2	550 ± 20 *	557 ± 14
Blood glucose at Week 4 (mg/dL, end of treatment)	93 ± 2	557 ± 13 *	407 ± 15 ###

Values are expressed as mean ± SEM. * *p* < 0.001 versus Control group; ### *p* < 0.01 versus Diabetes group.

**Table 2:** Histopathological degeneration and fibrosis scores in the control, diabetes, and diabetes + spermidine groups.

	Control Group	Diabetes Group	Diabetes + Spermidine Group
Endometrial gland degeneration score	0 [0–0]	3 [2–3] *	1 [0.75–1.25] ###
Endometrial stromal fibrosis score	0 [0–0]	2.5 [2–3] *	0 [0–1] ###
Ovarian follicle degeneration score	0 [0–0]	2 [1–2.25] *	1 [1–1.25] #
Ovarian stromal fibrosis score	0 [0–0]	1 [1–2] *	0.5 [0–1] #
Plasma AMH (ng/mL)	2.79 ± 0.2	1.29 ± 0.1 *	1.93 ± 0.1 ##

Variables are expressed using the median and interquartile range for histopathological scores and as mean ± SEM for plasma AMH. * *p* < 0.001 versus Control group; # *p* < 0.05, ## *p* < 0.01, ### *p* < 0.001 versus Diabetes group.

**Table 3:** Effects of diabetes and spermidine treatment on ovarian Nrf2, MDA, GSH, SOD, CAT, and GPx levels in rats.

Parameter (Unit)	Control	Diabetes	Diabetes + Spermidine
Ovary Nrf2 (pg/mg)	454.82 ± 21.83	257.99 ± 20.50 *	379.51 ± 20.15 ##
Ovary MDA (nmol/mg)	34.31 ± 3.64	80.77 ± 3.83 *	58.71 ± 4.07 ###
Ovary GSH (nmol/mg)	74.7 ± 3.2	31.9 ± 2.9 *	52.4 ± 2.9 ###
Ovary SOD (ng/mg)	9.67 ± 0.49	4.23 ± 0.42 *	7.32 ± 0.58 ##
Ovary CAT (ng/mg)	16.91 ± 0.68	5.98 ± 0.73 *	12.36 ± 0.44 ###
Ovary GPx (ng/mg)	17.27 ± 1.27	6.76 ± 0.64 *	12.73 ± 0.82 ###

Values are expressed as mean ± SEM. * *p* < 0.001 versus Control group; ## *p* < 0.01, ### *p* < 0.01 versus Diabetes group.

**Table 4:** Effects of diabetes and spermidine treatment on uterine Nrf2, MDA, GSH, SOD, CAT, and GPx levels in rats.

Parameter (Unit)	Control	Diabetes	Diabetes + Spermidine
Uterus Nrf2 (pg/mg)	421.97 ± 19.16	250.9 ± 22.91 *	345.5 ± 19.22 ##
Uterus MDA (nmol/mg)	38.12 ± 3.16	77.17 ± 3.28 *	60.84 ± 3.57 ##
Uterus GSH (nmol/mg)	76.83 ± 3.42	34.95 ± 3.37 *	57.55 ± 3.67 ###
Uterus SOD (ng/mg)	8.31 ± 0.42	3.63 ± 0.47 *	5.87 ± 0.52 ##
Uterus CAT (ng/mg)	13.50 ± 0.73	4.11 ± 0.40 *	9.05 ± 0.70 ###
Uterus GPx(ng/mg)	14.98 ± 0.61	4.69 ± 0.40 *	10.88 ± 0.81 ###

Values are expressed as mean ± SEM. * *p* < 0.001 versus Control group; ## *p* < 0.01, ### *p* < 0.01 versus Diabetes group.

**Table 5:** Effects of diabetes and spermidine treatment on ovarian and uterine LC3, Beclin-1, and TGF-β levels in rats.

Parameter (Unit)	Control	Diabetes	Diabetes + Spermidine
Ovary LC3 (ng/mg)	51.8 ± 2.6	24.0 ± 2.4 *	38.2 ± 1.8 ##
Ovary Beclin-1 (ng/mg)	5.6 ± 0.3	3.1 ± 0.2 *	4.2 ± 0.2 #
Ovary TGF-β (pg/g)	0.52 ± 0.05	1.25 ± 0.08 *	0.82 ± 0.06 ##
Uterus LC3 (ng/mg)	36.5 ± 2	21.1 ± 1.6 *	31.2 ± 2.2 ##
Uterus Beclin-1 (ng/mg)	4.3 ± 0.2	2.3 ± 0.2 *	3.6 ± 0.2 ###
Uterus TGF-β (pg/g)	0.79 ± 0.08	1.58 ± 0.09 *	1.10 ± 0.09 ##

Values are expressed as mean ± SEM. * *p* < 0.001 versus Control group; # *p* < 0.05, ## *p* < 0.01, ### *p* < 0.01 versus Diabetes group.

## Data Availability

The data presented in this study are available on request from the corresponding author.
